# Supplementary material for: A Qualitative Assessment of the Essential Health and Nutrition Service Delivery in the Context of COVID-19 in Bangladesh: The Perspective of Divisional Directors
Source: Healthcare (Basel). 2022 Aug 25;10(9):1619. doi: 10.3390/healthcare10091619 (PMC9498609; doi:10.3390/healthcare10091619)
Supplement: Supplementary file 1 [file healthcare-10-01619-s001.zip › healthcare-1887688-supplementary.pdf]

Supplementary Material

Nodes and subnodes used for the analysis and examples of significant quotes

Table S1. Codification of Service Delivery

**General aspects about service delivery**		
**Node**	**Sub node**	**Examples**
Not attending health services due to fear of getting infected with COVID-19	-Feeling safer at home	-Delivering a baby at home safer than at the clinic (Chittagong)<br>-Did not take children to get vaccinated due to fear (Barisal)
	-Health workers as carriers of the virus	-People would not go to the clinics fearing that they would be infected by health care providers (Barisal)
	-Stigmatization	- Some health-care providers faced stigmatization because they worked in hospital: "The staff, specifically women working in the health facilities, also faced emotional abuse for being healthcare workers and working in the COVID-19 pandemic situation in a hospital" (Dhaka)
	-Misinformation affected service utilization	-Perceptions about COVID-19 being spread in clinics increased fear and affected utilization (Barisal)<br>-Families who needed hospitalizations (i.e., delivery) often requested early discharge (Sylhet)<br>-Wrong communications exacerbated the notion that hospitals and clinics were only for COVID-19 (Barisal)<br>-Receiving misleading information through social media affected decisions about accessing services, a social media campaign was put in place (Barisal)
	-Difficulties in counterbalancing socially constructed perceptions	-There was generalized panic that affected health services delivery, it was difficult because there were no mechanisms to address this (Sylhet)
	-Low mortality reduced fear	-Seeing low mortality rates, reduced fear and help re-starting health care services use (Rajshahi)
Disruptions in service delivery	-Services focused mainly for emergencies	-During the worse months (March-April), most services were disrupted (all Divisions)
	-Government discouraged unnecessary visits	-Government suggested avoiding unnecessary visits to clinics and, when possible, encouraged use of telemedicine (i.e., online, phone consultation) (Sylhet, Dhaka)
	-Different types of services disrupted	-Supplementation during pregnancy (Barisal)<br>-Lack of in-person examinations during pregnancy (Khulna)

		-Immunizations, including outreach activities commonly performed, and shortages of vaccines (Rangpur, Khulna)<br>-Reduction in contraception recipients (there was no shortages of contraceptives, the problem was more focused on the delivery (Rangpur)<br>-Diabetes (which was often managed through the phone and self-management such as glucose monitoring) (Dhaka)<br>-Tuberculosis vaccination and diagnosis (Khulna)
	-Some instructions made it harder to getting services	-Instruction to check women close to delivery for COVID-19 (even if they were asymptomatic) (Chittagong)
	-Challenges of available health workers	-Many health workers were infected or died, this increased the pressure for the other health workers (Khulna)<br>-Those providers 55 and older, with comorbidities or pregnant had flexible tasks and increased workload for others (Khulna)
	-Affections on outreach activities	-No door-to-door activities reduced outreach (Barisal)<br>-Activities in houses and courtyard meetings were disallowed to enforce measures of social distancing (Barisal, Rangpur)<br>- During the critical period, community clinics provided regular services and health complexes continued treatments. They implemented schedule reductions: Health clinics reduced daily schedule (9-3) and Health assistants worked 3 out of 5 days (Khulna)<br>- Door-to-door visits and outreach sessions were cancelled during first months of lockdown. They resumed "when the death rate was slowing down in this division" (Dhaka)<br>- In-person motivation activities were substantially reduced compared to normal times (Mymensingh)
	-Challenges and alternative collaborations	-Private clinics and NGOs didn't come forward to provide services until later, when collaboration was achieved (most Divisions)<br>-It was problematic to distribute contraceptives, but drug stores were always open, although products cost (Khulna)
Health professionals looking how to provide services beyond clinics and adapting medical alternatives	-Telemedicine	-Services provided through telemedicine (as directed in governmental guidelines) (Dhaka)<br>- They used a help hotline, "Shashthya Batayan", where people call for a consultation with the doctor and to get information (Dhaka)<br>- Tele consultation increased a lot. "We became highly dependent on tele-consultation and telemedicine; this worked when we had better connectivity" (Khulna)

		- Teleconsultations were encouraged (Chittagong)
	-Other online services (i.e., zoom)	-Problems of connectivity and zoom solved by providers paying for these services out of pocket (Khulna)<br>-Facebook pages of clinics with doctors' phones so patients could reach them (Mymensingh)<br>-Use of Zoom not only to provide services but to coordinate service-related activities between providers (Mymensingh)
	-Mobile phones helped	-"Most people have access to mobile phones and they can contact the numbers of community health care workers… mobile phones helped a lot" (Mymensingh)<br>-Telephone numbers of doctors were circulated in the community. "Most people now have access to telephone, they somehow contacted doctors when they were in need" (Sylhet)
Delay in healthcare seeking linked to contextual factors	-Movement restrictions linked to lockdown rules	-Restrictions included travel bans, limiting transportation for healthcare seeking (Chittagong)<br>-Special transportations arrangements were needed to get medical supplies moving to different areas (Rajshahi)
	-Economic effects of the lockdowns	- Lockdowns produced economic hardship for many, leading to face difficulties in aspects such as buying medicines. Hence, role of community workers in distributing medicines was fundamental (Dhaka)<br>-Lockdown increased the risk of losing jobs, which increased internal migration (i.e., people employed elsewhere in the country returning home), as well as the risk of contagion (Sylhet)
Getting back to more stable service provision	-COVID-19 rules for service provision	-Strict mask wearing and explicit COVID-19 rules at clinics and hospitals helped reduced fear and increase utilization (Dhaka)
	-Outreach measures	-Outreach measures such as mobile immunization campaigns were launched (with support from NGOs) (Dhaka)<br>-Lack of fairs and interactive events might have enlarged the perceptions that hospitals were for COVID (Barisal)
	-Targeting groups who were known to had been affected	-WHO and UNICEF helped identifying children who missed immunizations (Chittagong)
	-Difficulties in service provision	-Not easy to maintain protocols in health facilities that have infrastructure not designed for this (i.e., inadequate access to water) (Barisal)<br>-Setting isolation units reduced the space for routine care (Rajshahi)<br>-Hospital reconversion has been difficult (Khulna)

		-COVID-19 as an extra task, without extra human resources (Sylhet)
	Collaboration with other actors	-Coordination with NGOs to help getting services back (Sylhet)
**Specific aspects about financing service delivery**		
Protective equipment (PE)	-Initially arrived late and with low quality	-PE arrived late, and this placed health care professionals at risk, doctors and other workers had to buy their own PE (Rangpur)<br>-Issue was not lack of budget, but time (i.e., PE arriving late) (Chittagong)<br>-Initial PE was of low standard, doctors bought their own (Dhaka)
	-Re-budgeting and donations	-Slowly budgets considered equipment at the upazila level (Rajshahi) and allocation of resources was set for hand sanitizers, gloves, etc. (Chittagong)<br>-NGOs increasingly donated PE (Chittagong)<br>-They did not have funds for PPE. Received them from the Central Medical Stores (Khulna)
Inflexible budgets for human resources	-Human resources gap needed alternative resources	-Despite the need, there was no support. An alternative was paid volunteers (Chittagong)
Technology	-Paying new technological needs	-Costs of new technological needs were addressed by the office (Dhaka)<br>-While the MIS Department provided support and orientation, practical issues were often solved through personnel money (Chittagong)<br>- They had to pay for the internet (Sylhet)<br>- CHWs occasionally had to cover costs of tele-consultations (Barisal)
**Specific aspects about guidelines**		
"Keep providing all services"	-Guidelines to keep proving all services	-Cleaning, social distancing, temperature, history of illness, mask wearing (Chittagong, Khulna)<br>-Encourage online treatment (Sylhet)<br>- "Most of the guidelines and instructions helped us"
	-Communication	-Regular meetings with the Directorate to discuss challenges and follow-up of guidelines (Rajshahi)<br>-WhatsApp groups with all Directors to help communications about decisions (Rajshahi)
**Specific aspects about human resources**		
Health professional	-Need for specific human resources	-Experts on occupational safety (Barisal)<br>-No public expert in the Division, no virologist (Chittagong)

workforce during COVID		-More medical technologists needed (Sylhet)
	-Important support from NGOs after lockdown	-Help training service providers (Barisal)<br>-Provide basic clinic and preventive services in urban public health clinics (Dhaka)
	-Not enough human resources to provide all services	-Lack of human resources was always an issue but it became more salient with COVID (Rangpur)<br>-New centers (i.e., alternative sites) were put together without extra staff (Chittagong)<br>-Lack of human resources also affected community services (Khulna)<br>-"Workload has increased as never before… leads to swamped work" (Chittagong)
	-Workload has been exhausting	-Health professionals working with devotion and determination (Khulna)<br>- Health professionals "superhuman beings" (Khulna)<br>-Needed to face unexpected situations such as death bodies brought to clinics (Mymensingh)<br>-Burden of health professions during the worst part of the crisis related to not seeing their loved ones, a mental health issue. Technology was a booster (Khulna)
	-Demands for better pay	-In November, "they actually went on strike" in different parts of the country to demand better pay and work conditions (Dhaka)<br>-Government offered incentives of 1-month bonus for their extra effort, but did not arrive (Khulna)
Administrative aspects in human management	-Divisions could not recruit	-No authority at the divisional office to recruit people during the emergency (Mymensingh)<br>-Lack of autonomy for recruiting needed personnel (Rangpur)<br>-Some personnel whose task was linked to outreach could not performed the job due to COVID restrictions and were not paid (Khulna)<br>-Vacant positions unable to be filled (Chittagong)
Pre-COVID strengths of the personnel	-Grassroot experience	-Health workers worked with community leaders to distribute information about COVID and search for active cases (Dhaka)<br>-It was also feasible to work with religious leaders to help with education, risk reduction of transmission during funerals, and outreach (Dhaka)
**Specific aspects about training and technology**		
Government encouraged the	-Technology support from third parties	-Technology support came from agencies like UNICEF (Khulna)

use of telemedicine		-Support from the MIS Department in zoom, telemedicine and online services (Barisal)<br>-Support from the MIS Department in installing online platforms and orientation about digital systems (Dhaka)
Uses of technology	-Support of technology during the pandemic	-Mobile phones have helped a lot as a contact mechanism with community health workers for consultation (Mymensingh)<br>-Facebook accounts were set for each clinic, which allowed communicating with patients via messenger (Chittagong)
	-Positive long-term effect in the use of technology	-Increased use of online services such as registration of vaccinations online and follow-up of pregnant women via e-registration (Chittagong)<br>-Most people now have a mobile phone that can be used to receive health services and health information (Barisal)<br>-Mobile phones from health providers were put at visible public places for patients to be able to call them (Chittagong)<br>-Use of media facilitated administration and meetings (Dhaka)
Trainings related to service delivery	-During the initial months of the pandemic	-Training in the use of protective equipment (Barisal)<br>-Training about keeping patients in isolation and testing (Mymensingh)
	-When services resumed	-How to resume outreach activities (Mymensingh)

Table S2. Codification of communication and community outreach

**General aspects about communication and community outreach**		
**Node**	**Sub node**	**Examples**
Adaptations during pandemic	Modifications	-In Sylhet "we discouraged people from attending health facilities and motivated community services through counselling".<br>-Traditional social gatherings and door-to-door visits are against COVID-19 guidelines. In Mymensingh, fear amongst CHW decreased until June, when they saw elders were not getting infected and not dying as often even if they were.<br>- Social distancing was protective but hard to keep for CHW, especially in social gatherings (Khulna).<br>-In Rangpur, localized and communication catch-sup campaigns were conducted to recover vaccination coverage with door-to-door outreach campaigns and phone calls and texting.<br>- They posted in Mymensingh doctor´s phone numbers from each upazila on Facebook. "It was effective to request treatment."
**Specific aspects about guidance**		

Node	Sub node	Examples
Communication with population	Messages	- Authorities agreed on messages and guidelines to be distributed by posters and leaflets (Rajshahi)<br>- Messages to community were not adapted (Chittagong). Conversely, NGOs and UN agencies adapted messages to local dialects, for example, in "Rohingya camps and Chittagong hill tracts."<br>- A consistent message in Barisal, Mymensingh, Rangpur, and Khulna was to avoid and postpone pregnancies: "We asked people not to get pregnant during COVID and promoted 15-20 slogans, selected by the Directorate's information and communication team".
	Tools	- In Rangpur mobile phones played an "important role." Official work was also done via Facebook.<br>-Community health workers in Barisal relied on text messages and phone calls<br>- In Chittagong and Barisal, they used a van to run awareness programs<br>- Sylhet circulated doctor´s phone numbers in the community. "We discouraged patients from going to the hospital and doctors provided services by phone."
	Misinformation	- In Rangpur social media "created confusion... People believed any news and welcome negative news…We told CHW not to believe and spread incorrect news."<br>- In Chittagong, Rajshahi, Sylhet and Mymensingh, they were unable to stop misinformation, affecting both service providers and seekers: "There was misinformation encouraging unscientific practices to prevent COVID."<br>-In Barisal "Lots of people falsely thought hospitals provided COVID-treatment only."
Communication between health providers	Guidelines	- Guidance was centered on COVID prevention in Rangpur<br>- Guidelines were mostly with COVID info, but in Sylhet they added contraceptives and family. They handed flyers for unwanted pregnancies with slogans.<br>- An instruction that pregnant women with signs of labor had to present a covid test within 5 days, even if asymptomatic, generated "chaos and additional trauma" in Mymensingh. Some women were sent back without results. The instruction then corrected that those deliveries should not be delayed in asymptomatic patients when tests were unavailable. Still, "women faced difficulties".
	Motivation	- In Barisal communication "between levels helped reduce fear, gain confidence and solve problems".

		- In Dhaka constant communication helped boost mental health and motivation
**Specific aspects about financing**		
**Node**	**Sub node**	**Examples**
Resources	Modifications	-Additional budget comes from Directorate and Civil Surgeon. Chittagong received funds for catch-up vaccination events.<br>- CHW informed the community in Mymensingh there was not a shortage of contraceptives supply
**Specific aspects about human resources**		
**Node**	**Sub node**	**Examples**
Community health workers (CHW)	Roles	-In Khulna, CHW played a "vital role" mobilizing and motivating people to get essential services. They visited households with pregnant women and children under 5 years to collect updates on health status and provide covid information.<br>- In Barisal, CHW were key for "setting up localized communication, catch-up campaigns, […and] immunization campaigns"<br>-The Rajshahi community trusts CHW and it helped when they encouraged immunization sessions, updates, and info sessions. They even communicated by mobile phone (most have one).
Partnerships	NGOs	- While government staff was busy with covid management, CHW from NGOs in Rangpur helped with coordination and communication of awareness programs and preventing unwanted pregnancies.<br>- In Sylhet "we always worked in close coordination with NGOs (i.e., Marie Stopes) […] We kept supplies and they collect contraceptives from family planning offices."
	Religious institutions	- Instructions in Chittagong underscored the involvement of Imams to raise covid awareness and encourage service utilization.<br>- Motivated service via the Imams in mosques because in Barisal "people continued to go to mosques defying the government´s instructions on social gathering".
**Specific aspects about training and technology**		
**Node**	**Sub node**	**Examples**
Technology	Information systems	- In Barisal, there was "center level guidance at each upazila for communication, remote follow-up, and flexible reporting due to connectivity problems… This was helpful."<br>- Information systems were used an expanded and perceived as helpful; in Dhaka "A database software called 'Gorbhobotir Aina' (roughly translated as pregnant woman's mirror) has been created to implement the Kapasia model in reducing maternal mortality."

Table S3. Codification of Surveillance and Service Monitoring

**Specific aspects about human resources**	
Supervision	**-** No in-persons supervision during first months because offices closed so in Sylhet, Rajshahi, and Chittagong they turned to monthly virtual meetings.<br>**-** Physical monitoring not possible so they turned to virtual meetings in Dhaka; they would have been more successful if started earlier on. Monitoring was conducted via quarterly supervision meetings.
**Specific aspects about financing**	
Laboratory Shortages	- In Chittagong, 6 districts have lab machines to examine blood tests in 40 minutes. They combined COVID and TB testing. Machines were not ready to be used; they used only 2 at Sadar hospital. They also need lab technicians for COVID-19 testing. There is no virologist or public health expert in the division.
**Specific aspects about training and technology**	
Monitoring systems	- In Barisal, disease surveillance depended on passive reporting of health facilities, which was reduced during the pandemic.<br>- In Khulna and Mymensingh, they developed a “system” to monitor field-level family planning workers, services, and locations. Results show missed routine checks and perceived reductions in ANC, but they do not have the data.
